# Functional Screen for microRNAs Suppressing Anchorage-Independent Growth in Human Cervical Cancer Cells

**DOI:** 10.3390/ijms23094791

**Published:** 2022-04-26

**Authors:** Angelina Huseinovic, Annelieke Jaspers, Annina P. van Splunter, Hanne Sørgård, Saskia M. Wilting, Dorian R. A. Swarts, Ida H. van der Meulen, Victor W. van Beusechem, Renée X. de Menezes, Renske D. M. Steenbergen

**Affiliations:** 1 Amsterdam UMC Location Vrije Universiteit Amsterdam, Pathology, De Boelelaan 1117, 1081 HV Amsterdam, The Netherlands; a.huseinovic@amsterdamumc.nl (A.H.); a.jaspers@amsterdamumc.nl (A.J.); a.vansplunter@amsterdamumc.nl (A.P.v.S.); hanne.sorgard@gmail.com (H.S.); 2 Cancer Center Amsterdam, Imaging and Biomarkers, 1081 HV Amsterdam, The Netherlands; 3 Department of Medical Oncology, Erasmus University MC Cancer Institute, Dr. Molewaterplein 40, 3015 GD Rotterdam, The Netherlands; s.wilting@erasmusmc.nl; 4 College of Life Sciences, University of Amsterdam, Science Park 904, 1081 HV Amsterdam, The Netherlands; d.r.a.swarts@uva.nl; 5 Amsterdam UMC Location Vrije Universiteit Amsterdam, Medical Oncology, De Boelelaan 1117, 1081 HV Amsterdam, The Netherlands; ih.vandermeulen@amsterdamumc.nl (I.H.v.d.M.); vw.vanbeusechem@amsterdamumc.nl (V.W.v.B.); 6 Cancer Center Amsterdam, Cancer Biology and Immunology, 1081 HV Amsterdam, The Netherlands; 7 Netherlands Cancer Institute—Antoni van Leeuwenhoek Hospital (NKI-AvL), Biostatistics Unit, Plesmanlaan 121, 1081 HV Amsterdam, The Netherlands; r.menezes@nki.nl

**Keywords:** ultra-low attachment, miRNA screen, anchorage-independent growth, soft-agar assays, cervical cancer

## Abstract

The progression of anchorage-dependent epithelial cells to anchorage-independent growth represents a critical hallmark of malignant transformation. Using an in vitro model of human papillomavirus (HPV)-induced transformation, we previously showed that acquisition of anchorage-independent growth is associated with marked (epi)genetic changes, including altered expression of microRNAs. However, the laborious nature of the conventional growth method in soft agar to measure this phenotype hampers a high-throughput analysis. We developed alternative functional screening methods using 96- and 384-well ultra-low attachment plates to systematically investigate microRNAs regulating anchorage-independent growth. SiHa cervical cancer cells were transfected with a microRNA mimic library (n = 2019) and evaluated for cell viability. We identified 84 microRNAs that consistently suppressed growth in three independent experiments. Further validation in three cell lines and comparison of growth in adherent and ultra-low attachment plates yielded 40 microRNAs that specifically reduced anchorage-independent growth. In conclusion, ultra-low attachment plates are a promising alternative for soft-agar assays to study anchorage-independent growth and are suitable for high-throughput functional screening. Anchorage independence suppressing microRNAs identified through our screen were successfully validated in three cell lines. These microRNAs may provide specific biomarkers for detecting and treating HPV-induced precancerous lesions progressing to invasive cancer, the most critical stage during cervical cancer development.

## 1. Introduction

Normal epithelial cells are dependent on proper cell-to-cell and cell-to-matrix adhesions (“anchors”) for differentiation and proliferation. Loss of these adhesions results in aberrant integrin signaling and subsequent induction of anoikis (detachment-induced cell death) [1,2]. Similarly in vitro, primary cells need to attach to the surface of the culturing dish to survive and proliferate. Cancer cells, on the other hand, are capable of growing in the absence of attachment to the surface, known as anchorage-independent growth representing a hallmark of cancer. The acquisition of anchorage-independent growth during human papillomavirus (HPV)-induced carcinogenesis has been recognized as a critical event associated with the progression to tumorigenic cells [3]. Infection with high-risk HPV types has been associated with anogenital (cervical, vulvar, vaginal, anal, penile) and oropharyngeal cancers. The HPV-induced anogenital cancers are preceded by well-recognized precancerous lesions, referred to as intraepithelial neoplasia. These lesions have a heterogeneous nature and only a subset has the potential to progress to cancer [4]. Recognition of the advanced lesions with a high risk of progression to cancer is essential to improve clinical management of affected patients, and to prevent overtreatment of patients with precancerous lesions having a low cancer risk [4]. Our previous studies showed that a subset of precancerous lesions was characterized by (epi)genetic changes, such as chromosomal aberrations, DNA methylation, and differential microRNA (miRNA) expression profiles, and that their accumulation reflected a higher risk of progression to invasive cancer [3,5,6]. Using an in vitro model system of HPV16 and HPV18 transfected keratinocytes, closely mimicking HPV-induced carcinogenesis, it was found that the progression of HPV-immortalized cells towards anchorage-independent growth was likewise associated with an accumulation of (epi)genetic changes in the host cell [7,8,9]. Comparison of anchorage-dependent immortal cells to anchorage-independent cells showed the most prominent global change in miRNA expression, indicating that altered miRNA expression was associated with the acquisition of anchorage independence during malignant transformation [8].

miRNAs represent an abundant class of small non-coding RNAs that regulate gene expression post-transcriptionally by targeting mRNAs for degradation, destabilization, or inhibition of translation [10]. They are involved in the regulation of a variety of important cellular processes including cell development, differentiation, apoptosis, and signal transduction. miRNAs are aberrantly expressed in a variety of tumors and play a significant role in tumor development and carcinogenesis by dysregulation of oncogene and tumor suppressor gene expression [11,12]. We and others have identified a number of miRNAs associated with the development of HPV-induced cancers and the acquisition of anchorage independence in cervical cells, such as miR-129-2-3p and miR-137 [8,13,14,15,16,17,18,19,20]. We propose that the identification of miRNAs functionally involved in the acquisition of anchorage independence, a critical step towards carcinogenicity, might provide a rewarding strategy in the search for the development of novel treatment strategies and molecular biomarkers enabling improved early cancer detection.

The golden standard to study anchorage independence is the soft agar assay [8,21]. However, the laborious nature of this assay hampers a high-throughput analysis. The availability of ultra-low attachment (ULA) cell culture plates, in which cell adherence to the surface is inhibited by a special coating forcing cells to stay in suspension, provides new opportunities for high-throughput functional analysis. The feasibility of this approach is supported by a comparative study in which it was found that cell growth in low-attachment conditions was highly similar to growth in soft-agar assays [22,23]. Here we employed ULA plates as an alternative to soft agar assay to perform an unbiased functional screen on the role of miRNAs in anchorage-independent growth of HPV-positive SiHa cervical cancer cells.

## 2. Results

### 2.1. Functional Screen for miRNA Suppressors of Anchorage-Independent Growth

**Figure 1:**
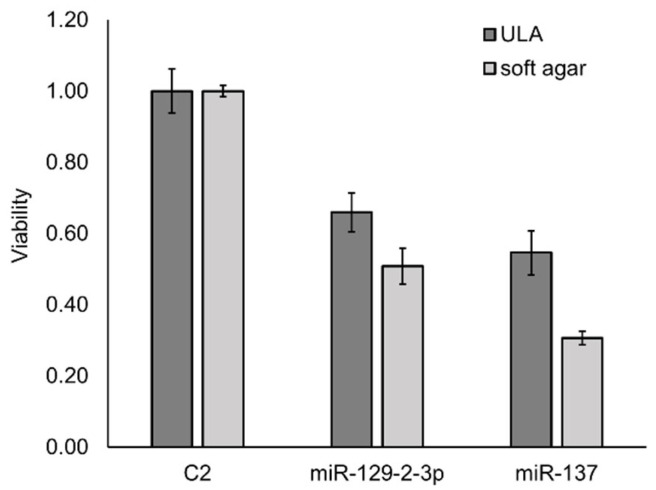
ULA plates show similar growth reduction compared to soft-agar assays. Bar graph demonstrating comparable results for a cell viability assay using ULA plates and colony counts in a soft-agar assay for SiHa cells ectopically expressing miR-129-2-3p or miR-137, relative to negative control C2. The standard error was calculated based on at least two measurements.

To determine whether ULA plates are suitable for high-throughput functional analysis of anchorage-independent growth of SiHa cervical cancer cells, we first assessed whether the results obtained using ULA plates were comparable with the soft agar assay outcomes. SiHa cells were transfected with miR-129-2-3p and miR-137 mimics, two miRNAs that were previously validated as suppressors of anchorage-independent growth [8]. Both miRNAs showed a very similar growth-inhibiting effect when comparing colony counting in soft-agar assays to cell viability measurements after culturing cells in ULA plates (Figure 1). This indicates that cell viability measurements upon growth on ULA plates provide a promising alternative for the classical soft-agar assay.

**Figure 2:**
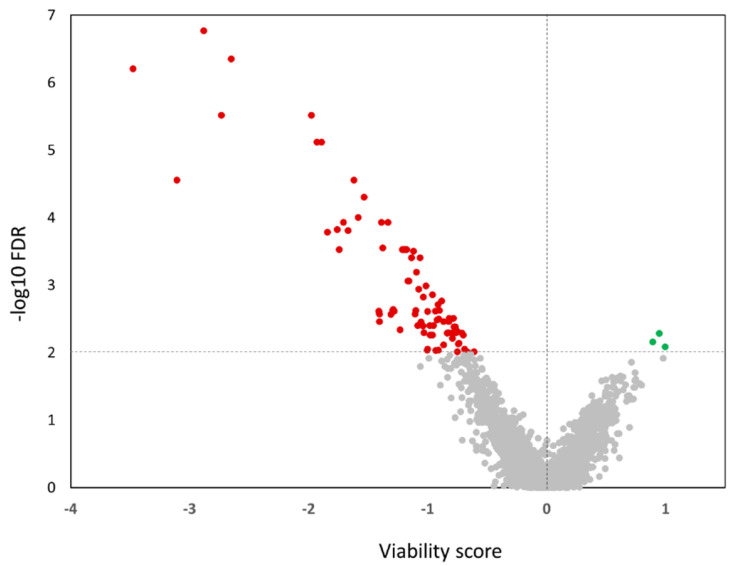
Volcano plot of viability scores in SiHa cells. The x-axis specifies the difference in viability scores, and the y-axis specifies the negative logarithm to the base 10 of the FDR. The horizontal dashed red line indicates the selection threshold: FDR < 0.01. miRNA hits with decreased and increased viabilities are marked red and green, respectively.

Next, we proceeded with a genome-wide functional screen on SiHa cells using the miRIDIAN miRNA mimic library comprising 2019 miRNA mimics. The data were log-2 transformed and visualized in boxplots to select the most suitable positive control for the data normalization. The strongest effect on cell viability was measured for UBB siRNA followed by miRNA mimics miR-137 and miR-129-2-3p, as shown in Supplementary Figure S1A. Cell viability data from the miRNA mimics library yielded mostly values fitting between the negative control C2 and the positive control miR-137 (Supplementary Figure S1B). Therefore, these were used to calculate viability scores, representing normalized log2-values relative to the controls. The negative control was centered at zero (no change in viability) and the positive control (miR-137) at -1. Based on a false discovery rates (FDR) <0.01 we identified 87 miRNAs significantly affecting the growth of SiHa cells in ULA plates, 84 of which were decreasing growth (values <0), and 3 were increasing the growth (values >0) (Figure 2). As expected, miR-137 and miR-129-5p in the library were also identified amongst the 84 miRNAs reducing growth on ULA plates. The complete data set is presented in Supplementary File S1.

### 2.2. Independent Validation and Selection of miRNAs Specifically Reducing Anchorage-Independent Growth

**Figure 3:**
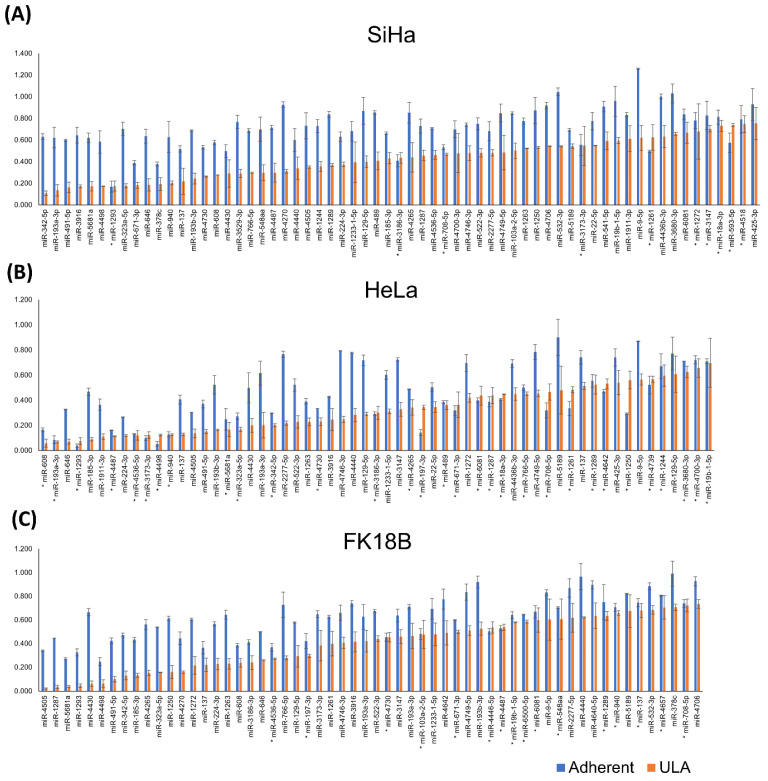
Validated miRNA mimics reducing growth on ULA plates. Cell viability results in adherent (blue) and ULA (orange) plates of validated miRNAs in SiHa (**A**), HeLa (**B**), and FK18B (**C**) cells. miRNAs annotated with * show no difference between adherent and ULA according to the set threshold. miRNAs without * show higher growth reduction in ULA plates than adherent plates and are referred to as the “ULA-norm-Ad”. The error bars represent the standard errors of two measurements.

To determine which of the 84 miRNAs were specifically reducing anchorage-independent growth, a smaller scale screen with 384-well ULA and adherent plates was performed on SiHa and HeLa cervical cancer cells and HPV18-transformed human keratinocytes, FK18B cells. First, transfection conditions were further optimized for each individual cell line (Supplementary File S2, Figures S2 and S3). In these validation screens we aimed to identify miRNAs that reduce growth on ULA plates but not on adherent plates to exclude miRNAs reducing growth unrelated to anchorage independence. We identified miRNA mimics group with reducing growth in ULA plates, named “ULA”, and a subgroup of these representing miRNA mimics with significantly higher growth reduction in ULA plates when compared to the adherent plates, named “ULA-norm-Ad”. In SiHa cells, reduction of growth in ULA plates could be confirmed for 63 out of 84 (75%) miRNA mimics of which 53 (63%) were classified as “ULA-norm-Ad”. Analysis of HeLa and FK18 cells showed similar results with 59 miRNA (70%) miRNA mimics in “ULA” and 29 (35%) in “ULA-norm-Ad” group for HeLa, and 58 miRNAs (69%) in “ULA” and 42 (50%) in “ULA-norm-Ad” for FK18B cells (Figure 3).

**Figure 4:**
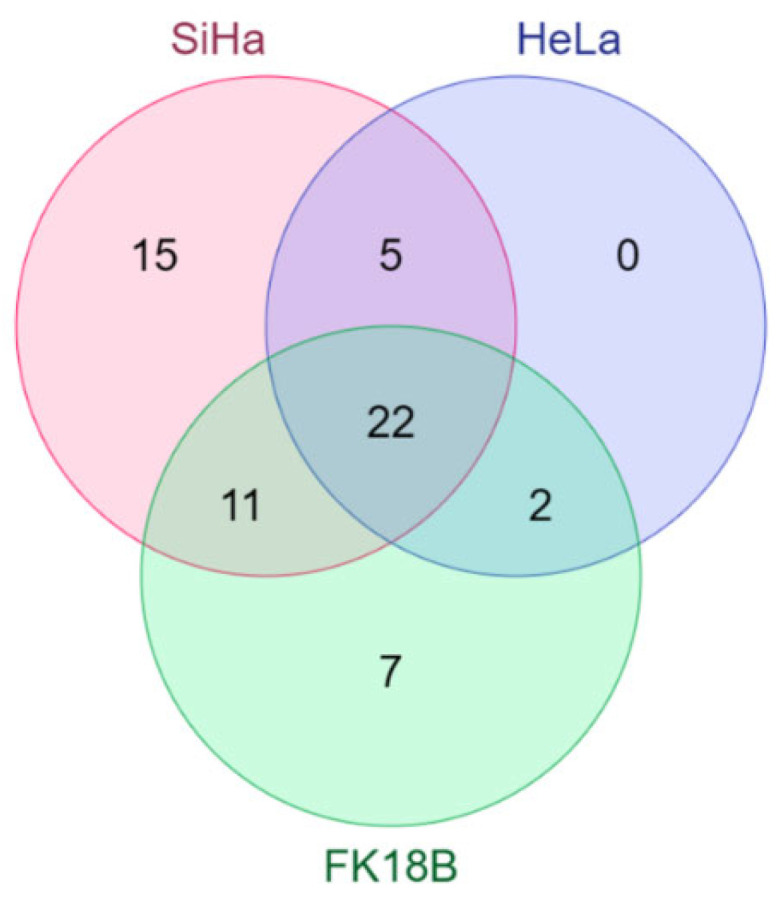
Overlapping hits of miRNAs from the “ULA-norm-Ad” group between SiHa, HeLa, and FK18B cells.

Comparison of miRNAs specifically affecting anchorage-independent growth (“ULA-norm-Ad”) identified 40 miRNA that overlap between at least two cell lines: 22 between all three cell lines, 5 between SiHa and HeLa, 11 between SiHa and FK18B, and 2 between HeLa and FK18B. Fifteen, 1 and 7 miRNAs were unique in SiHa, and FK18B cells, respectively (Table 1, Figure 4). The complete lists of all miRNAs, including the normalized cell viability data, the classification to “ULA” and “ULA-norm-Ad” groups and unvalidated miRNAs, is presented in Supplementary File S3.

**Figure 5:**
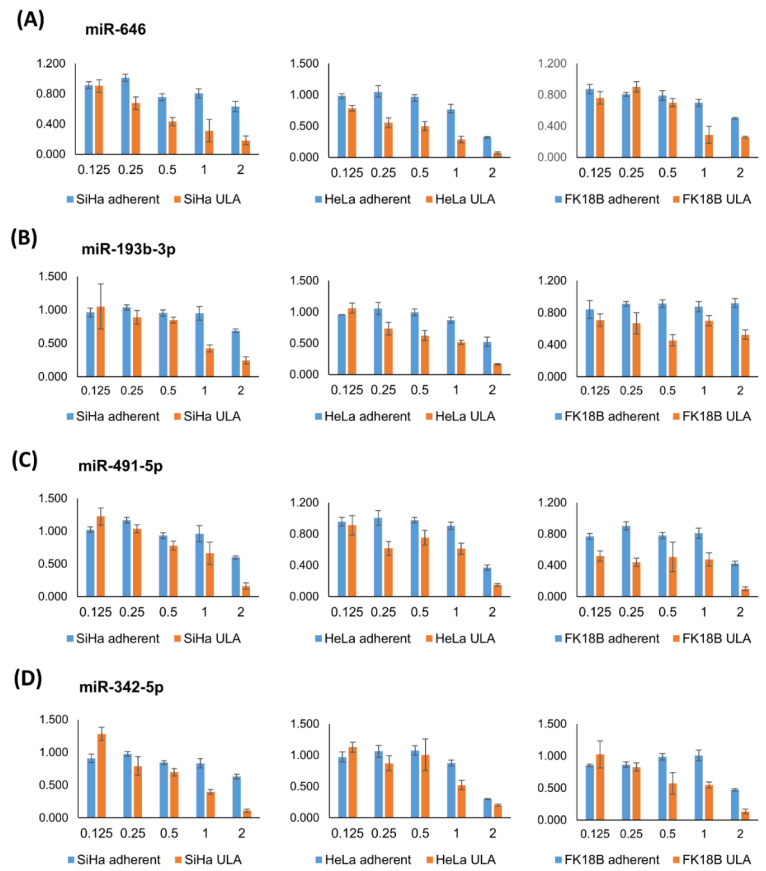
Dose-dependent effect of miRNA mimic transfection. SiHa, HeLa and FK18B cells were transfected with 1, 0.5, 0.25 and 0.125 nM of miR-646 (**A**), miR-193-3p (**B**), miR-491 (**C**) and miR-342-5p (**D**). The values represent normalized cell viabilities relative to negative control C2, in adherent (blue) and ULA (orange) plates. The values of the validation screen using 2 nM concentrations are also shown as a reference. The error bars represent the standard error of two measurements.

Upon validation, a strong growth reduction in both adherent and ULA plates was observed for a subset of miRNAs. To determine if a potential specific effect on anchorage-independent growth was masked using a too high miRNA mimic concentration, further miRNA dilutions were tested. Hereto, four miRNAs, miR-646, miR-193b-3p, 491-5p, and miR-342-5p, all causing a strong growth reduction in all three cell lines in both ULA and adherent plates, were transfected at lower miRNA mimic concentrations: 1, 0.5, 0.25 and 0.125 nM. As shown in Figure 5, lowering the miRNA mimic concentration yielded conditions in which a decrease in cell viability was only seen in ULA plates. These results confirm that the difference between the cell viability in ULA and adherent plates can be used as a selection criterion even when a significant effect in adherent plates is observed.

### 2.3. Review of the Literature on Validated miRNAs

To obtain further evidence for a role in cancer of the 40 validated miRNAs (“ULA-norm-Ad”) that were overlapping in at least 2 cell lines, we performed a literature search with a focus on their relation to cancer. Interestingly, 21 miRNAs were described as tumor suppressors in a variety of cancers, 10 of which were also tumor suppressors in cervical cancer, i.e., miR-129-5p, miR-137, miR-491-5p, miR-185-3p, miR-532-3p, miR-646, miR-1287, miR-224-3p, miR-22-5p, and miR-193b-3p (Table 2). Eight miRNAs were described as oncomiRs and were found to be upregulated in several cancers, including three in cervical cancer: miR-3147, miR-766-5p, miR-224-3p. Altogether, the majority of the identified hits are described in some relation to cancer, and mostly as tumor suppressors.

## 3. Discussion

Anchorage independent growth in a critical step in cancer development with miRNAs playing a crucial role in the regulation of this process. Here we describe a functional screen with 2019 miRNA mimics to identify suppressors of anchorage independence in SiHa cervical cells using ultra-low attachment plates. After thorough validation in both adherent and ULA plates in SiHa, HeLa, and FK18B cell lines, we confirmed 40 miRNAs as novel suppressors of anchorage independence in cervical cancer.

The current gold-standard assay for determining anchorage-independent growth, the soft-agar assay is a laborious technique that is not easily adaptable for use in genome-wide high-throughput screens. Previous attempts to screen for anchorage-independent growth are therefore scarce, such as performing a soft-agar assay in a 96-well format using shRNA libraries to study tumor suppressive miRNAs in colorectal cancer [153], or in a 384-well format to screen for anticancer compounds in human lung cancer cells [154]. Although the soft agar assay can be performed in a smaller high-throughput format, this is practically challenging and requires quick handling to prevent premature agar solidification, especially when handling large libraries. Ultra-low attachment plates that recently became available in 96- and 384-well format were previously validated as an alternative for the soft-agar assay [22,23] and also enabled us to develop a high-throughput method to determine relevant suppressors of anchorage independence in cervical cancer cells. Differences between these two methods lie mainly in the harshness of low attachment conditions. While in the soft agar assay cells are prevented from attachment to the surface and each other, on ULA plates, although prevented to attach to the surface, cells are free to migrate, to attach to each other, and to grow as spheroids. The benefit of making conditions less stringent is that we can measure differences in cell viabilities after 3 days compared to 3 weeks in soft agar assay where cells need to form visible colonies to be counted. Whilst developing this functional screening method, several other studies have undertaken a similar approach, such as drug discovery screens with head and neck cancer cells [155,156]. To our knowledge, the current study is the first miRNA mimic screen for suppressors of anchorage-independent growth in cervical cancer cell lines. Earlier functional screens using miRNA mimics in cancer research were conducted in 2D cultures to study tumor-suppressive miRNAs directly [157,158,159,160], or following exposure to radiation [161,162]. Other screens focused on miRNAs regulating NF*κ*B-signaling [163], HER2-signaling [164], or adenovirus propagation in prostate cancer [165].

While for the primary functional screen with the complete miRNA mimic library (n = 2019) we used only ULA plates, adherent plates were introduced as a reference for further validation experiments. This allowed selection of miRNAs affecting specifically anchorage-independent growth. Following further optimization of the screening protocol, we were able to significantly improve transfection efficiency, simplify the workflow by switching from forward to reverse transfection, and reduce materials. Furthermore, analysis of lower concentrations of miRNA mimics for those that resulted in a strong growth reduction showed a dose-dependent biological effect of these miRNAs on cell viability. Therefore, to obtain most optimal conditions and for future reference, it would be useful to test lower concentrations on all identified hits during validations.

Since cancer is a heterogeneous disease, using different cell lines to identify overlapping hits might, by inference, increase the odds of finding universal targets and biomarkers. Therefore, introducing more (pre) cancerous cell lines during the validation step will probably be beneficial. The validity of our functional screen is supported by the fact that the control miRNAs miR-137 and miR-129-5p, known to suppress anchorage-independent growth in SiHa cells [8], were also identified as overlapping hits between SiHa, HeLa, and FK18B cells. These two miRNAs are also described by others to act as tumor-suppressors in cervical cancer [24,25,26,27,28]. Several other miRNAs that were identified, such as miR-491-5p, miR-1287, miR-193b-3p, miR-532-5p and miR-22-5p, miR-185-3p, and miR-646 have been described as tumor suppressors in cervical cancer [55,56,60,65,72,76,82,83]. Moreover, the majority of the identified miRNAs were described as acting as tumor-suppressors in a variety of cancers. We also identify miRNAs not yet described in the literature in relation to cervical cancer or cancer in general, making them interesting candidates for further analysis. Future studies will include target identification and further studies on their biological role.

To conclude, we successfully developed and validated a high-throughput functional screen using 96-and 384-well ULA plates to study the effect of miRNAs on anchorage-independent growth. The practical knowledge obtained allows for additional functional screens using newly extended miRNA, CRISPR-Cas9, siRNA libraries or drug libraries as well as for testing of other cell lines.

## 4. Materials and Methods

### 4.1. Cell Culture

Cervical carcinoma cell lines SiHa (RRID:CVCL_0032) and HeLa (RRID:CVCL_0030) were obtained from the American Type Culture Collection (Manassas, VA, USA) and authenticated by STR testing using the Powerplex16 System (Promega, Madison, WI, USA). The cells were cultured in Dulbecco’s Modified Eagle Medium (DMEM) (Thermo Fisher Scientific, Waltham, MA, USA) supplemented with 10% fetal bovine serum (FBS), 100 U/mL penicillin, 100 µg/mL streptomycin, and 2 mmol/L L-glutamine (all Thermo Fisher Scientific). FK18B cells passage p251, anchorage-independent human keratinocytes transfected with full length HPV18, were developed and characterized as described previously [7,8] and cultured in keratinocyte-SFM medium (KGM) supplemented with human EGF, pituitary gland extract, 100 U/mL penicillin, 100 µg/mL streptomycin, and 292 µg/mL L-glutamine (all Thermo Fisher Scientific). All cell lines tested negative for mycoplasma prior to experiments.

### 4.2. Growth Comparison between ULA Plates and Soft Agar Assay upon miRNA Mimic Transfection

For transfection in 96-well ULA plates (Corning, New York, NY, USA), 10000 cells SiHa cells per well were seeded and after 1 h transfected with complexes of 20 nM mimics and 0.1 µL DharmaFECT 1 (Horizon Discoveries, Cambridge, UK) in DMEM medium without FBS and antibiotics. The cells were grown for 4 days at 37 °C, 5% CO_2_ and cell viabilities were measured using CellTiterBlue assay (Promega). Soft-agar assays were performed as described previously [166]. Briefly, SiHa cells were plated in 6-well tissue culture adherent plates (Corning) and transfected after 24 h with 30 nM of miRNA mimics miR-129-2-3p and miR-137 and non-targeting negative control C2 (Horizon Discovery, C-301063-01, C-300604-07, and CN-002000-01, respectively) using DharmaFECT 2 (Horizon Discovery) as transfection reagent. Next day, the cells were harvested by trypsinization and 5000 cells were plated in 6-well plates mixed with medium containing soft-agarose. Colonies were formed and counted approximately 3 weeks after the transfection. All experiments were performed at least in duplicate.

### 4.3. High-Throughput miRNA Mimic Screen

miRIDIAN miRNA mimic library (version 19, containing 2019 miRNA mimics) was purchased at Horizon Discovery. The miRNA mimic screen was performed in triplicate using twenty-six 96-well ULA plates (Corning, #3474) per replicate. Three positive controls and one negative control were included on each plate: miR-129-2-3p and miR-137 (Horizon Discovery, C-301063-01 and C-300604-07, respectively) as positive controls for anchorage-independent growth, siGENOME siRNA against human Ubiquitin B gene (UBB) (Horizon Discovery, M-013382-01), an essential gene, as positive control for transfection efficiency, and a non-targeting control C2 (Horizon Discovery, CN-002000-01) as negative control. On day 1, SiHa cells were seeded at a concentration of 10,000 cells per well and transfected with 20 nM miRNA mimic or siRNA using 0.4 μL DharmaFECT 4 transfection reagent (Horizon Discovery) per well. On day 4, cells were assayed for cell viability by adding 20 μL of CellTiter-Blue Reagent (Promega) and measuring fluorescence at 540 nm excitation and 590 nm emission wavelengths using a Tecan Infinite F200 reader (Tecan Group, Männedorf, Switzerland).

### 4.4. Screen Data Processing and Normalization

Fluorescent cell viability read-outs were processed and normalized for inter- and intra-plate variations using Rscreenorm (v1.0), a statistical method recently implemented as an R package [167], R (v3.6.3). One plate (replicate 2, plate 7) failed to be loaded by the robot before measurement and had to be omitted. The data were read into R using the package cellHTS2 [168] and the cell viability values were log2-transformed. Boxplots of log2-viability measurements were made per plate of each replicate to visually investigate the plate effect (the same growth pattern per individual well across all plates). From the three positive controls, the most suitable control for the screen analysis was chosen based on its viability read-out being closest to the upper values (highest lethality) found in the sample data set. Subsequently, log2-transformed data were re-expressed as the lethality score relative to the median values of the positive and negative control using the formula: Lethality score = (miRNA mimic-negative control C2) / (positive control - negative control C2), in which values near zero represent viability similar to that of the negative controls, and those near 1 similar to that of the positive control. Lethality scores were computed to correct for the plate effects within each plate, and quantile normalization was performed to correct for differences in the distribution of lethality scores between different replicates. Subsequently, the normalized lethality scores of miRNA mimics were compared to those of the negative control using an empirical-Bayes linear regression model, part of R package limma [169]. Then, false discovery rates (FDRs) were calculated using empirical-Bayes linear regression [170] and miRNA mimics with FDR< 0.01 were considered to have a significant lethality score yielding a list of miRNA candidates that potentially affect anchorage-independent growth. Since in our experiment miRNA overexpression can also result in an increased growth (negative lethality score), we re-expressed the results as viability scores by multiplying lethality scores with −1: values below and above zero represent then reduced and increased viability, respectively. 

### 4.5. Validation of miRNA Mimic Candidates

#### 4.5.1. Transfection Protocol

Candidate miRNAs with FDR < 0.01 were further validated using reverse transfection in 384-well ULA and adherent (normal tissue culture) plates (Corning #4588 and #3764, respectively). Optimization of the conditions is described in Supplementary File S3. Following optimal conditions, 10 µL of 8 nM miRNA mimics and 10 µL of 0.04 µL DharmaFECT 4 (Horizon Discoveries) per well were mixed in OptiMEM medium (Thermo Fisher Scientific) and incubated for 1.5 h. For testing of lower concentrations 10 µL of 4, 2, and 1 nM of miRNAs mimics were used. Subsequently, 500 cells resuspended in 20 µL of culture media were added to each well, resulting in a final volume of 40 µL and miRNA mimic end concentration of 1, 0.5, 0.25, and 0.125 nM. Each transfection was performed in duplicate. As a positive control, siGENOME siRNA for the human UBB gene (Horizon Discoveries) was used. As negative controls, miRNA mimic non-targeting controls C1 and C2 (Horizon Discovery, CN001000-01 and CN-002000-01, respectively), and a panel of 16 miRNAs that showed no effect on growth in previous research were used, (Supplementary File S2). Cells were assayed for cell viability 3 days post transfection by adding 20 μL CellTiter-Glo reagent (Promega) per well and the luminescence was measured using Tecan Infinite F200 reader (Tecan Group).

#### 4.5.2. Data Analysis

For each cell line, SiHa, HeLa, and FK18B, and per ULA and adherent plate, the average of two cell viability measurements was normalized with the average of the panel of 16 negative controls and standard errors were calculated. After normalization, the values close to 1 represented cell viability similar to the negative controls, and the values lower than 1 represented reduced cell viability. Using the normalized cell viability data, we first determined miRNA mimics reducing growth in ULA plates: we chose values that were lower than 1, then calculated the average and standard deviation (stdev), and set the threshold below average + stdev. The list of chosen hits with reduced cell viability in ULA plates were named “ULA”. Subsequently, we used these values to determined miRNA mimics that significantly reduced growth in ULA plates compared to adherent plates, making the cell viability read-out specific for anchorage-independent growth. For this, we calculated the difference in cell viability between adherent and ULA plates (adherent-ULA) per miRNA mimic, chose the values above 0, and calculated stdev for all the differences. The threshold was then set above one stdev. The resulting list of candidates is called “ULA-norm-Ad”. ULA-norm-Ad lists of each cell line were intersected and overlapping miRNAs affecting anchorage independent growth in at least two of three cell lines were determined as final hits. For testing of lower miRNA concentrations, the averages of two measurements were normalized to negative control C2 for adherent and ULA plates. Standard errors were calculated according to the general rule for propagation of standard errors for divisions and subtractions.

## Figures and Tables

**Table 1:** Overlapping miRNAs reducing growth in SiHa, HeLa and/or FK18B cells.

Gene ID	Overlap 3 Cell Lines	Gene ID	Overlap 2 Cell Lines
hsa-miR-129-5p	SiHa, HeLa, FK18B	hsa-miR-22-5p	SiHa, HeLa
hsa-miR-137	SiHa, HeLa, FK18B	hsa-miR-103a-2-5p	SiHa, HeLa
hsa-miR-185-3p	SiHa, HeLa, FK18B	hsa-miR-425-3p	SiHa, HeLa
hsa-miR-193a-3p	SiHa, HeLa, FK18B	hsa-miR-1911-3p	SiHa, HeLa
hsa-miR-193b-3p	SiHa, HeLa, FK18B	hsa-miR-4436b-3p	SiHa, HeLa
hsa-miR-224-3p	SiHa, HeLa, FK18B	hsa-miR-323a-5p	SiHa, FK18B
hsa-miR-491-5p	SiHa, HeLa, FK18B	hsa-miR-342-5p	SiHa, FK18B
hsa-miR-522-3p	SiHa, HeLa, FK18B	hsa-miR-378c	SiHa, FK18B
hsa-miR-532-3p	SiHa, HeLa, FK18B	hsa-miR-608	SiHa, FK18B
hsa-miR-646	SiHa, HeLa, FK18B	hsa-miR-766-5p	SiHa, FK18B
hsa-miR-1233-1-5p	SiHa, HeLa, FK18B	hsa-miR-1250	SiHa, FK18B
hsa-miR-1263	SiHa, HeLa, FK18B	hsa-miR-1287	SiHa, FK18B
hsa-miR-2277-5p	SiHa, HeLa, FK18B	hsa-miR-3680-3p	SiHa, FK18B
hsa-miR-3916	SiHa, HeLa, FK18B	hsa-miR-4270	SiHa, FK18B
hsa-miR-4265	SiHa, HeLa, FK18B	hsa-miR-4498	SiHa, FK18B
hsa-miR-4430	SiHa, HeLa, FK18B	hsa-miR-5681a	SiHa, FK18B
hsa-miR-4440	SiHa, HeLa, FK18B	hsa-miR-1272	HeLa, FK18B
hsa-miR-4505	SiHa, HeLa, FK18B	hsa-miR-3147	HeLa, FK18B
hsa-miR-4706	SiHa, HeLa, FK18B		
hsa-miR-4746-3p	SiHa, HeLa, FK18B		
hsa-miR-4749-5p	SiHa, HeLa, FK18B		
hsa-miR-5189	SiHa, HeLa, FK18B		

**Table 2:** Literature search of 40 identified “ULA-norm-Ad” hits in relation to their role in cancer.

Gene ID	Function/Expression	Type of Cancers	#
hsa-miR-137	Tumor suppressor Anchorage-independent growth reduction	**Cervical** [24,25,26,27,28], melanoma [29], acute lymphoblastic leukemia [30], pituitary adenoma [31], prolactinomas [32], pancreatic [33], endometrial [34], colon [35], glioblastoma [36], melanoma [37], bladder [38], liver [39], pancreatic [40], and other	305
hsa-miR-129-5p	Tumor suppressor	**Cervical** [8,41,42,43], gastric [44,45], prostate [46], lung [47,48], colon [49], breast [50], pancreatic [51], liver [52], bladder [53], many other	159
hsa-miR-491-5p	Tumor suppressor	**Cervical** [54,55,56], colon [57], osteosarcoma [58], gastric [59], many others	49
hsa-miR-193b-3p	Tumor suppressor	**Cervical** [60], lung [61], ovarian [62], gastric [63], acute myeloid leukemia [64]	43
hsa-miR-532-3p	Tumor suppressor Downregulated	**Cervical** [65], colon [66], lung [67], lymphoma [68], prostate [69], tongue [70], breast [71] and other	36
hsa-miR-646	Tumor suppressor	**Cervical** [72], breasts [73], lung [74], gastric [75], many others	31
hsa-miR-185-3p	Tumor suppressor Downregulated	**Cervical** [76], prostate [77], colon [78], osteosarcoma [79], breast [80], nasopharyngeal [81], and other	21
hsa-miR-1287	Tumor suppressor Downregulated	**Cervical** [82,83], breast [83,84,85,86,87,88], liver [87], colon [89], pancreatic [90], lung [88] and other	
hsa-miR-22-5p	Tumor suppressor Downregulated	**Cervical** [65,91,92,93], osteosarcoma, prostate, lung [93], and other	16
hsa-miR-378c	Tumor suppressor Prognostic marker	**Cervical** [94], gastric [95], Wilms tumor [96], head and neck [97]	12
hsa-miR-193a-3p	Tumor suppressor	Lung [98,99,100], esophageal [101], liver [102], renal [103], osteosarcoma [104], colon [105], many others	107
hsa-miR-608	Tumor suppressor	Prostate [106], bladder [107], lung [108]	80
hsa-miR-342-5p	Tumor suppressor	Breast [109,110,111], ovarian [112], colon [113], osteosarcoma [114], neuroblastoma [115,116]	27
hsa-miR-4270	Tumor suppressor	Lung [117,118], liver [119]	10
hsa-miR-103a-2-5p	Tumor suppressor	Prostate [120], tongue [121], colon [122]	5
hsa-miR-4749-5p	Tumor suppressor	Glioblastoma [123]	
hsa-miR-1911-3p	Tumor suppressor	Lung [124]	
hsa-miR-1250	Tumor suppressor	Non-Hodgkin’s lymphoma [125]	
hsa-miR-323a-5p	Tumor suppressor	Neuroblastoma [115,116]	
hsa-miR-3680-3p	Tumor suppressor	Esophageal [126]	
hsa-miR-1272	Tumor suppressor	Prostate [127]	
hsa-miR-5189	Prognostic marker	Childhood B-cell acute lymphoblastic leukemia [128]	
hsa-miR-4430	Downregulated Prognostic marker	Ovarium [129]	
hsa-miR-224-3p	OncomiR	**Cervical** [130,131], osteosarcoma [132], lung [133]	15
hsa-miR-766-5p	Upregulated, OncomiR	**Cervical** [134], promotes other cancers	6
hsa-miR-3147	Upregulated, OncomiR Prognostic marker	**Cervical** [135], vulvar [136]	2
hsa-miR-522-3p	OncomiR	Lung [137,138], colon [139]	12
hsa-miR-425-3p	Upregulated Prognostic marker	Liver [140], gastric [141], prostate [142], lung [143,144], colon [145]	11
hsa-miR-1233-1-5p	Upregulated	Renal [146,147] esophageal [148]	
hsa-miR-3916	Upregulated, OncomiR	Melanoma [149], urothelial tract [150]	
hsa-miR-4440	Upregulated Prognostic marker	Colon [151]	
hsa-miR-4505	Upregulated Prognostic marker	Gastric [152]	
**No (cancer related) publications:** hsa-miR-2277-5p, hsa-miR-4498, has-miR-5681a, has-miR-4436b-3p, hsa-miR-4706, hsa-miR-1263, hsa-miR-4265, hsa-miR-4746-3p			

# Number of cancer-related publications. For each miRNA we performed search in Pubmed using the word “cancer” and the number of listed publications is noted in the last column. miRNAs are sorted based on their relevance to cervical cancer following with the relevance to other cancers and the number of publications starting with tumor suppressive miRNAs and following with oncomiRs.

## Data Availability

The data that support the findings of this study are available in the Supplementary Materials of this article.

## Supplementary Materials

The following supporting information can be downloaded at: https://www.mdpi.com/article/10.3390/ijms23094791/s1.

## Abbreviations

human papillomavirus (HPV), microRNA (miRNA), ultra-low attachment (ULA), false discovery rates (FDR).
