# Supplementary material for: Functional Screen for microRNAs Suppressing Anchorage-Independent Growth in Human Cervical Cancer Cells
Source: Int J Mol Sci. 2022 Apr 26;23(9):4791. doi: 10.3390/ijms23094791 (PMC9100801; doi:10.3390/ijms23094791)
Supplement: Supplementary file 1 [file ijms-23-04791-s001.zip › Supplementary File S2.pdf]

Supplementary File S2

Further screen optimization for the validation in three cell lines

After successfully performing screens on SiHa cells with 96-well ULA plates, the screen conditions were further optimized in order to increase the transfection efficiency, simplify the process, and reduce the materials. The most important aspects are summarized in **Table S1**. A switch from forward to reverse transfection was made thereby reducing robot use. The pipetting robot is only needed for library preparation and not for transfection, for which an automatic dispenser can be used. The cell viability assay was changed from CT-Blue to CT-Glo 3D (Promega), which is quicker to perform and more suitable for measuring of cell viability of spheroids that form on ULA plates. Further reduction of used materials such as medium and DharmaFECT 4 (Horizon Discovery) was obtained by using 384-wells instead of 96-well plates. Black 384-well plates with transparent bottom facilitated measurement of the luminescent signal. The use of Opti-MEM medium instead of DMEM w/o serum to make miRNA mimic/ DharmaFECT 4 complexes increased transfection efficiency approximately ten times. Combined with a reduced final volume (100 µl to 40 µl) this resulted in a 25 times reduction in use of miRNA mimics. Furthermore, as reference we also introduced adherent plates in the screen to make a distinction between miRNAs affecting anchorage-independent growth (only on ULA plates) versus cell growth in general. The crucial parameters for efficient transfection with no or reduced toxicity that needed to be individually determined for each cell line were miRNA mimic concentration, DharmaFECT 4 amount and amount of cells per well.

Table S1. Additional screen optimization in 384-well ULA plates

**Parameters**	**Original screen**	**Optimized screen**
Cell viability assay	100 µl of CT-Blue	20 µl of CT-Glo 3D
Format plates	96-well, transparent	384-well, black
Type plates	ULA	ULA + AD
Transfection	Forward	Reverse
Robot	Yes	No
Mimics end concentration	20 nM	2 nM
Total volume	100 µl	40 µl
DharmaFECT 4/well	0.4 µl	0.03-0.04 µl
Transfection medium	DMEM w/o serum	Opti-MEM
Amount of cells/well	10 000	500

Transfection optimization

For the screen validation experiments, the optimal transfection conditions in ULA plates for SiHa, HeLa and FK18B cells were determined using different concentrations of miRNAs and DharmaFECT 4. The transfections were performed as described in Material and methods. For FK18B cells we tested at 2, 5, 10 and 20 nM miRNA mimic concentration combined with 0.03-0.06 µl of DharmaFECT 4 per well, and compared the transfection efficiency when using Opti-MEM or KGM medium to make transection mixes. We used positive control siRNA-UBB and negative miRNA mimic control C1 and compared the cell viabilities with the cells that grew only in medium to assess the transfection toxicity. In Figures S2A and S2B, it is shown that for FK18B cells using Opti-MEM a 10x higher transfection efficiency is obtained, and that 0.03-0.04 µl of DharmaFECT 4 showed the least toxicity at any used concentration.

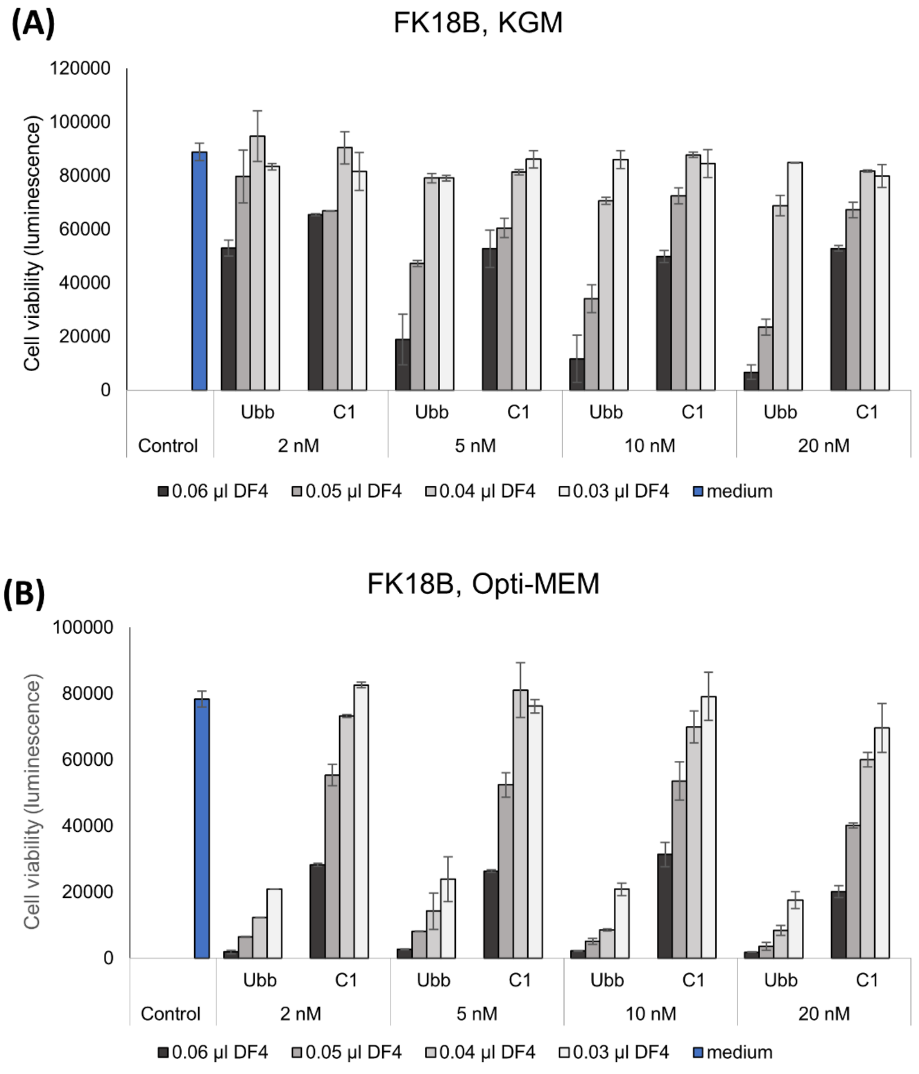

Figure S2. Reverse transfection optimization. FK18B cells were transfected with different concentrations miRNA mimics (2, 5, 10 and 20 nM, shades of gray) and amounts of DharmaFECT 4 (DF4) per well (0.06, 0.05, 0.04 and 0.03 µl, black and different shades of grey) in KGM **(A)** and Opti-MEM **(B)** as transfection media. Cell viabilities were measured three days post transfection using CT-Glo assay. siRNA against human UBB gene was used as positive control and non-targeting miRNA control C1 as negative control. As control for transfection toxicity we compared cell viabilities of C1 transfected cells to the cells grown in only medium (blue). The error bars represent standard errors of two measurements.

For SiHa and Hela cell transfection optimization, we used Opti-MEM to make transfection complexes, since it resulted in a much higher efficiency on FK18B cells. We tested miRNA at concentrations 1, 2, 5 and 10 nM, and DharmaFECT 4 at 0.03-0.06 µl. SiHa cells were clearly sensitive to DF4, with a dose-dependent decrease of cell viability in negative control C2 transfected cells. DharmaFECT 4 amount 0.04 µl in combination with any of the tested miRNA concentrations resulted in acceptable toxicity in SiHa cells with a good transfection efficiency (Figure S3A). For HeLa cells, DharmaFECT 4 was not toxic at tested amounts, and 0.05-0.06 µl of DharmaFECT 4 showed the best efficiency with any of the tested miRNA concentrations (Figure S3B).

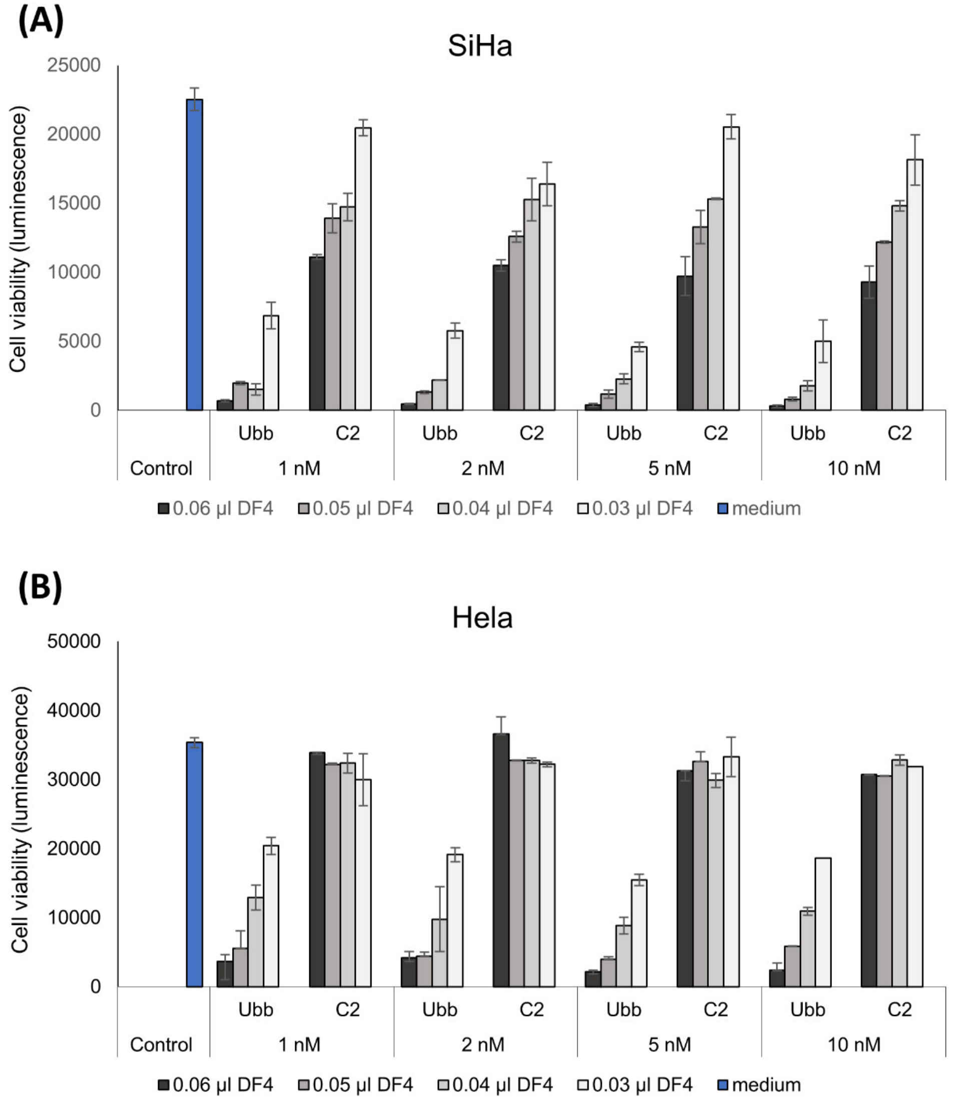

Figure S3. Transfection efficiency optimization of SiHa and Hela cells. Different concentrations miRNA mimics (2, 5, 10 and 20 nM) and amounts of DharmaFECT 4 (DF4) per well (0.06, 0.05, 0.04 and 0.03 µl, black and different shades of gray) were used to transfect SiHa **(A)** and HeLa **(B)** cells. Cell viabilities were measured three days post transfection using CT-Glo assay. siRNA against human UBB gene was used as positive control and non-targeting control C2 as negative control. As control for transfection toxicity we compared cell viabilities of C2 transfected cells to the cells grown in only medium (blue). The error bars represent standard errors of two measurements.
