# Supplementary material for: Functional Screen for microRNAs Suppressing Anchorage-Independent Growth in Human Cervical Cancer Cells
Source: Int J Mol Sci. 2022 Apr 26;23(9):4791. doi: 10.3390/ijms23094791 (PMC9100801; doi:10.3390/ijms23094791)
Supplement: Supplementary file 1 [file ijms-23-04791-s001.zip › Supplementary Figure S1.pdf]

Supplementary Figure S1

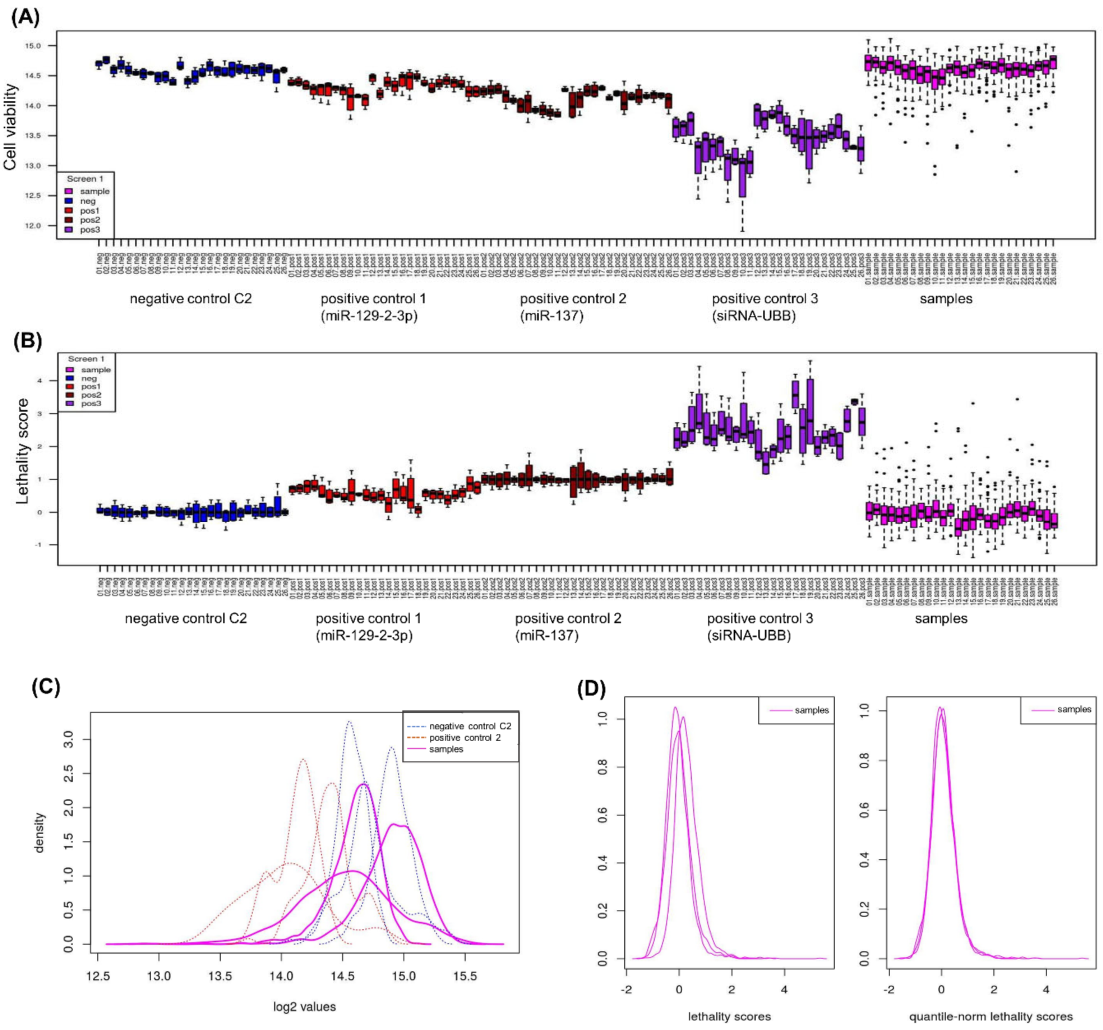

Figure S1. Plate normalization using Rscreenorm of cell viability read-outs of mimic-library-transfected SiHa cells. Boxplots of log2-viability measurements per plate of replicate 1 are shown. Every boxplot represents a summary of all measurements of one plate. Note that the number of controls per plate (n=16) is lower than the number of samples (approximately 78 per plate). (**A**) Cell-viability read-outs prior to normalization. (**B**) Lethality scores following normalization to the negative control C2 and positive control miR-137. Values above 1 represent miRNA mimics that have a stronger effect on cell viability than miR-137, while negative lethality scores represent miRNAs that stimulate cell viability when compared to the negative control. **(C)** Density plots of the log2-transformed viability values confirm the observation that library miRNAs' log2-viability values (magenta) overlap with both the negative control 2 (blue) as well as the positive control miR-137 (red). **(D)** Quantile-normalization of lethality scores of three replicates
